# International Medical Congress

**Published:** 1887-01

**Authors:** 


INTERNATIONAL MEDICAL CONGRESS.

We are glad to know that the prospects for the next meeting of the International Medical Congress which is to be held in Washington next summer, are at present more bright than appeared possible at one time. The gross injustice done to the original committee by the actions of the American Medical Association at the New Orleans meeting of 1885 still exists in full force, and, we suppose, must remain so. The consequence is that the majority of the brightest lights of the American profession may take no part in the congress. This fact will cause many on the continent and in Great Britain to remain at home; and has also had a serious effect on a large portion of the masses of the profession in the United States and Canada.

Notwithstanding all these drawbacks it must be confessed that the new enlarged executive committee has done its work wonderfully well, and has thereby made great strides in gaining the confidence of the profession throughout the world. The names of many distinguished men, especially from Great Britain, France and Germany, have consented to act as officers with various sections. The venerable President-elect, Dr. Davis, of Chicago, is highly and deservedly respected by all sections of the profession; and was very well received at the recent meeting of the British Medical Association, where, without doubt, he gained many friends for the coming congress.

We sincerely hope that all parties will now unite in their endeavors to make the meeting an unqualified success. We would like to see the leaders of the profession in the United States act as a unit on this question. Let the present committee manage the executive business of the congress as its members think best—and they will probably do it well; at the same time let the ablest men of the United States unite in their endeavors to make the scientific proceedings a grand, indisputable success. In doing so they, and not the successful manipulators of the New Orleans vote, will carry off the honors; but they will, apart from any such selfish considerations, be acting as loyal and patriotic citizens and physicians in showing to the world what wonderful progress has been made in things medical by the American Medical Profession, and also in exhibiting those social qualities which make the practical and generous citizens of the United States the most charming and agreeable hosts in the world. —*Canadian Practitioner.*

